# Sarcosine is a prostate epigenetic modifier that elicits aberrant methylation patterns through the SAMe‐Dnmts axis

**DOI:** 10.1002/1878-0261.12439

**Published:** 2019-03-09

**Authors:** Vladislav Strmiska, Petr Michalek, Zuzana Lackova, Roman Guran, Sona Krizkova, Lucie Vanickova, Ondrej Zitka, Marie Stiborova, Tomas Eckschlager, Borivoj Klejdus, Dalibor Pacik, Eliska Tvrdikova, Claudia Keil, Hajo Haase, Vojtech Adam, Zbynek Heger

**Affiliations:** ^1^ Department of Chemistry and Biochemistry Mendel University in Brno Czech Republic; ^2^ Central European Institute of Technology Brno University of Technology Czech Republic; ^3^ Department of Biochemistry Faculty of Science Charles University Prague 2 Czech Republic; ^4^ Department of Paediatric Haematology and Oncology 2nd Faculty of Medicine Charles University University Hospital Motol Prague 5 Czech Republic; ^5^ Central European Institute of Technology Mendel University in Brno Czech Republic; ^6^ Department of Urology University Hospital Brno Brno Czech Republic; ^7^ Faculty of Medicine Masaryk University Brno Czech Republic; ^8^ Department of Pathology University Hospital Brno Czech Republic; ^9^ Department of Food Chemistry and Toxicology Technical University of Berlin Germany

**Keywords:** DNA methylation, Dnmts, epigenetics, prostate cancer, SAMe, sarcosine

## Abstract

DNA hypermethylation is one of the most common epigenetic modifications in prostate cancer (PCa). Several studies have delineated sarcosine as a PCa oncometabolite that increases the migration of malignant prostate cells while decreasing their doubling time. Here, we show that incubation of prostate cells with sarcosine elicited the upregulation of sarcosine N‐demethylation enzymes, sarcosine dehydrogenase and pipecolic acid oxidase. This process was accompanied by a considerable increase in the production of the major methyl‐donor *S*‐adenosylmethionine (SAMe), together with an elevation of cellular methylation potential. Global DNA methylation analyses revealed increases in methylated CpG islands in distinct prostate cell lines incubated with sarcosine, but not in cells of nonprostate origin. This phenomenon was further associated with marked upregulation of DNA methyltransferases (Dnmts). Epigenetic changes were recapitulated through blunting of Dnmts using the hypomethylating agent 5‐azacytidine, which was able to inhibit sarcosine‐induced migration of prostate cells. Moreover, spatial mapping revealed concomitant increases in sarcosine, SAMe and Dnmt1 in histologically confirmed malignant prostate tissue, but not in adjacent or nonmalignant tissue, which is in line with the obtained *in vitro* data. In summary, we show here for the first time that sarcosine acts as an epigenetic modifier of prostate cells and that this may contribute to its oncometabolic role.

**Abbreviations**
5‐Aza: 5‐azacytidine
AAS: atomic absorption spectrometry
*AR*: androgen receptor
BSP: bisulphited polymerase chain reaction
*CCND2*: cyclin D2
*CD44*: CD44 antigen
*CDKN2B*: cyclin‐dependent kinase inhibitor 2B
CMP: cellular methylation potential
DESI MSI: desorption electrospray ionization mass spectrometry imaging
DMGDH: dimethylglycine dehydrogenase
Dnmts: DNA methyltransferases
ESI‐QqTOF MS: electrospray ionization quadrupole–quadrupole time‐of‐flight mass spectrometry
FLD: fluorescence detection
*FOS*: Fos proto‐oncogene
GNMT: glycine *N*‐methyltransferase
ICC: immunocytochemistry
IHC: immunohistochemistry
*JUN*: Jun proto‐oncogene
MALDI‐TOF: matrix‐assisted laser desorption/ionization time‐of‐flight
PCa: prostate cancer
PIPOX: pipecolic acid oxidase
SAH: *S*‐adenosylhomocysteine
SAMe: *S*‐adenosylmethionine
SARDH: sarcosine dehydrogenase

## 1 Introduction

Chromatin structure defines the state in which genetic information in the form of DNA is organized (Toh *et al*., 2017). Recent advances in cancer research have shown that global changes in the epigenetic landscape are a hallmark of various types of malignant diseases, including prostate cancer (PCa) (Jeronimo *et al*., 2011; Jones and Baylin, 2002, 2007). Unlike DNA mutations, epigenetic changes induce conformational changes in the DNA double helix and modify transcription factor access to promoter regions upstream of coding sequences (Hanahan and Weinberg, 2011). Among the epigenetic modifications, DNA hypermethylation is one of the most common in PCa (Dobosy *et al*., 2007; Geybels *et al*., 2015; Hoque *et al*., 2005). Methylation is a result of the enzymatic transfer of a methyl group from the methyl‐donor *S*‐adenosylmethionine (SAMe) to the C5‐position of cytosine, while cytosines are mostly methylated when bound to guanines (Jeronimo *et al*., 2011). The hypermethylation of regions with a high G/C content (palindromic CpG islands) of tumour suppressor genes leads to their inactivation, which may represent an early event in PCa development (Valdes‐Mora and Clark, 2015).

Sarcosine (*N*‐methyl glycine) is a widely discussed noninvasive biomarker of the early stages of PCa (Khan *et al*., 2013; Sreekumar *et al*., 2009). Several studies have delineated sarcosine as a PCa oncometabolite that increases the migration of malignant prostate cells while decreasing their doubling time (Heger *et al*., 2016a). Sarcosine also induces PCa cell invasion and intravasation *in vivo* (Khan *et al*., 2013) and affects expression of genes involved in cell cycle regulation and apoptosis (Heger *et al*., 2016a). Despite the obvious importance of sarcosine in PCa progression, the molecular mechanisms of its action have not yet been fully elucidated.

**Figure 1:**
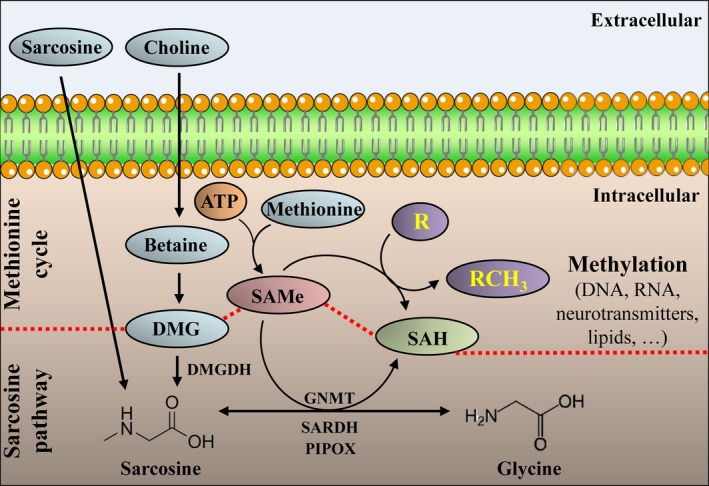
Schematic representation of the linkage between SAMe, SAH, sarcosine metabolic pathway and methionine cycle. Sarcosine can either be taken up or formed from dimethylglycine (DMG) by DMGDH (EC 1.5.8.4). Alternatively, sarcosine can be formed from glycine by GNMT (EC 2.1.1.20). Sarcosine can be N‐demethylated by SARDH (EC 1.5.8.3) or PIPOX (EC 1.5.3.1) to glycine with the latter providing the methyl‐donor, SAMe, in the methionine cycle. Finally, SAMe is demethylated into SAH, which is associated with the methylation of an acceptor (R) into RCH_3_. EC, enzyme classification number of the International Union of Biochemistry.

In its biochemical pathway, sarcosine is produced from glycine by glycine *N*‐methyltransferase (GNMT) or alternatively from dimethylglycine by dimethylglycine dehydrogenase (DMGDH) (Heger *et al*., 2016a). Conversely, sarcosine oxidative N‐demethylation yielding glycine is promoted by sarcosine dehydrogenase (SARDH) and pipecolic acid oxidase (PIPOX) (Cha *et al*., 2014). This process provides a methyl group that can be utilized for the methylation of homocysteine that completes a cycle in which the monocysteinyl moiety is converted sequentially from methionine to SAMe (Wilson *et al*., 2009). Consequently, SAMe can be demethylated to *S*‐adenosylhomocysteine (SAH) (Sibani *et al*., 2002). During this process, methyl groups are supplied for numerous transmethylation reactions, including the methylation of nucleic acids (the pathway is schematized in Fig. 1). It is a general fact that epigenetic and metabolic alterations in cancer cells are highly intertwined; however, despite the obvious linkage between sarcosine and methionine pathways, whether sarcosine metabolism can contribute to the hypermethylation of DNA remains unknown.

Therefore, the aim of this study was to investigate the relationship between sarcosine metabolism and molecules pivotal for methylation processes. For the first time, we showed that the incubation of prostate cells with sarcosine resulted in a complex response that involved the elevated formation of the methyl‐donor SAMe and increased global DNA methylation and stimulation of the expression of DNA methyltransferases (Dnmts) – enzymes responsible for the catalytic transfer of methyl groups from SAMe to DNA (van der Wijst *et al*., 2015). Moreover, we demonstrated that sarcosine was partially able to avert the inhibition of Dnmts induced by the hypomethylating agent 5‐azacytidine (5‐Aza) and that 5‐Aza inhibited sarcosine‐induced migration of prostate cells. These data showed that sarcosine metabolism is coupled to aberrant DNA methylation, which is considered one of the major hallmarks of cancer.

## 2 Materials and methods

### 2.1 Chemical compounds

All standards and other chemicals were purchased from Sigma‐Aldrich (St. Louis, MO, USA) with ACS purity, unless noted otherwise.

### 2.2 Cell lines and treatment rationale

Three human prostate cell lines were used in this study: (a) the PNT1A human cell line, established by immortalization of normal adult prostatic epithelial cells; the primary culture was obtained *postmortem* from the prostate of a 35‐year‐old male; (b) the 22Rv1 human PCa epithelial cell line, derived from a xenograft that was serially propagated in mice after castration‐induced regression and relapse of the parental, androgen‐dependent CWR22 xenograft; and (c) the LNCaP human cell line, established from an androgen‐sensitive metastasis located in the left supraclavicular lymph node in a 50‐year‐old Caucasian male. To evaluate the specificity of DNA methylation, we employed the following nonprostate cell lines: A2780 (ovarian cancer); MDA‐MB‐231 (triple‐negative breast cancer); and SH‐SY5Y and UKF‐NB‐4 (neuroblastoma). Except for UKF‐NB‐4 that was a kind gift from Eckschlager, cell lines used in this study were purchased from the Health Protection Agency Culture Collections (Salisbury, UK). The culture media used were as follows: Roswell Park Memorial Institute‐1640 (RPMI‐1640) for culturing PNT1A, 22Rv1, LNCaP and A2780 cells; Dulbecco′s Modified Eagle's Medium for culturing MDA‐MB‐231 and SH‐SY5Y cells; and Iscove′s Modified Dulbecco′s Medium for culturing UKF‐NB‐4 cells. For culturing, the media were supplemented with 10% fetal bovine serum and penicillin (100 U·mL^−1^) and streptomycin (0.1 mg·mL^−1^). The cells were maintained at 37 °C in a humidified incubator Galaxy 170R (Eppendorf, Hamburg, Germany) with 5% CO_2_. The treatment with sarcosine was initiated after the cells reached ~ 70–80% confluence. For administration, 1 μm sarcosine (sterile‐filtered, BioXtra product line) was used throughout the study. The rationale behind this decision was that this concentration was shown to stimulate a proliferation of prostate cells *in vitro* and *in vivo* (Heger *et al*., 2016b). As controls, we analysed the cells exposed to relevant volume of vehicle [sterile Milli‐Q water), Merck Millipore, Burlington, MA, USA) w/o sarcosine).

### 2.3 Quantification of sarcosine intracellular uptake

Intracellular sarcosine was quantified using a HPLC HP 1100 Series (Palo Alto, CA, USA) coupled with a fluorescence detector (FLD operating with λ_exc_ = 350 nm, λ_em_ = 450 nm). Upon incubation (due to expectation of a fast intracellular accumulation, the longest incubation time was 3 h), the cells were washed with PBS (5×) and extracted in a mixture of MeOH and 1 m acetic acid (80 : 20 v/v). Chromatographic separation and detection was performed after precolumn derivatization with *o*‐phthalaldehyde and fluorenylmethyloxycarbonyl chloride. The compounds were eluted with a linear upward gradient of mobile phases composed by acetonitrile/water (90 : 10 v/v) and 0.1 m ammonium formate in water.

### 2.4 Immunocytochemistry

For immunocytochemistry (ICC), cells were grown on eight‐well chamber slides and incubated with sarcosine (1 μm, 24 h). Then, cells were fixed with 4% paraformaldehyde for 15 min. After permeabilization with 0.3% Triton X‐100 in PBS for 3 min, the cells were blocked with 10% BSA in PBS and incubated with the indicated primary antibodies at 4 °C overnight. ICC was analysed with an EVOS FL Auto Cell Imaging System (Thermo Fisher Scientific, Waltham, MA, USA). Rabbit anti‐GNMT (1 : 500) and rabbit anti‐SARDH (1 : 200) were from Abcam (Camridge, UK); mouse anti‐DMGDH (1 : 500) and mouse anti‐PIPOX (1 : 200) were from Santa Cruz Biotechnology (Dallas, TX, USA); and mouse anti‐Dnmt1 (1 : 1000) was from Thermo Fisher Scientific. ICC was quantified using ImageJ (National Institutes of Health, Bethesda, MD, USA), analysing the intensity of 80–120 cells per group with subsequent background subtraction (analysis without using primary antibodies).

### 2.5 Extraction and quantification of SAMe and SAH

*S*‐adenosylmethionine and SAH were extracted in a mixture of MeOH and acetic acid [1 m, 80 : 20 (v/v)]. Briefly, 300 μL of solvent was added to the frozen cells followed by slow thawing on ice. The quantification of SAMe and SAH upon 2‐, 6‐, 12‐ and 24‐h incubation with sarcosine (1 μm) was performed using HPLC with electrospray ionization quadrupole–quadrupole time‐of‐flight mass spectrometer (HPLC‐ESI‐QqTOF MS) Maxis Impact (Bruker Daltonik GmbH, Bremen, Germany). The separation was performed on the C18 reverse phase column Phenomenex Kinetex EVO (Phenomenex, Torrance, CA, USA). As mobile phases, water with 0.1% (v/v) formic acid and MeOH with 0.1% (v/v) formic acid were used.

### 2.6 Global analysis of DNA methylation

DNA was extracted from cells incubated with sarcosine (1 μm, 24, 48 and 72 h) using the ExtractNow™ DNA Mini Kit (Minerva Biolabs, Berlin, Germany) according to the manufacturer's instructions and quantified at λ = 260 nm using a spectrophotometer, Infinite 200 PRO (Tecan, Mannedorf, Switzerland). The global DNA methylation analysis was performed using the commercial methylated DNA quantification kit (MDQ1 Imprint kit) in 96‐well plates according to the manufacturer's instructions. The readout was performed at 450 nm. The results are expressed upon subtracting background.

### 2.7 Bisulphite treatment of genomic DNA, bisulphite polymerase chain reaction (BSP) and sequencing

Two micrograms of genomic DNA was treated with sodium bisulphite using the Epitect Bisulphite Kit (Qiagen, Hilden, Germany), according to the manufacturer's instructions. After bisulphite conversion, DNA was amplified with specific primers designed using the MethPrimer (Li and Dahiya, 2002) and Bisulphite Primer Seeker 12S (Zymo Research, Irvine, CA, USA) with emphasis on the amplification of CpG islands in the promoters or CpG‐rich regions. The list of genes and sequences of primers used for BSP are provided in Table S1. All primers were confirmed to not amplify any nonbisulphited DNA. PCR products were analysed on 1% agarose gels, and the bands were purified with QIAEX II Gel Extraction Kit (Qiagen). The purified DNA was analysed by Sanger sequencing by SEQme company (Dobris, Czech Republic).

### 2.8 Analysis of intracellular spermine (Spm) and spermidine (Spd)

Polyamines (Spm and Spd) were analysed on an ion‐exchange chromatography AAA 400 (Ingos, Prague, Czech Republic) with a postcolumn derivatization by ninhydrin and two‐channel Vis detector (λ = 440 and 570 nm) (Cernei *et al*., 2016).

### 2.9 Quantification of the mobile pool of intracellular zinc

Intracellular free zinc was analysed according to a slightly modified protocol (Haase *et al*., 2006). Briefly, cells were grown in 96‐well plates up to 70–75% confluency and pretreated with sarcosine or zinc sulphate for 24 or 48 h. Then, the medium was removed, and the cells were loaded with 2.5 μm Zinpyr‐1 in loading buffer [10 mm 4‐(2‐hydroxyethyl)‐1‐piperazineethanesulfonic acid, pH 7.35, 120 mm sodium chloride, 5.4 mm KCl, 5 mm glucose, 1.3 mm calcium chloride, 1 mm magnesium chloride, 1 mm sodium dihydrogen phosphate and 0.3% BSA] and analysed on a Tecan Infinite M200 reader (Tecan) using λ_exc_ = 492 nm, λ_em_ = 527 nm.

### 2.10 Quantification of total intracellular zinc using atomic absorption spectrometry (AAS)

Prior to total zinc quantification, cells were washed with 10 μm ethylenediaminetetraacetic acid to remove extracellular bound zinc and then digested in nitric acid (65% v/v) and hydrogen peroxide (30% v/v). Total zinc was analysed using the graphite furnace AAS 280Z (Agilent Technologies, Santa Clara, CA, USA) with Zeeman background correction.

### 2.11 Isolation of RNA and qRT‐PCR

High pure total‐RNA isolation kit (Roche Life Science, Indianapolis, IN, USA) was used for isolation of cellular RNA of cells incubated with sarcosine (24 h). The medium was removed, and samples were washed twice with 5 mL of ice‐cold PBS. Cells were scraped off, transferred to clean tubes and centrifuged at 20 800 ***g*** for 5 min at 4 °C. After that, lysis buffer was added and RNA isolation was carried out according to manufacturer's instructions. RNA (500 ng) was transcribed using Transcriptor First Strand cDNA Synthesis Kit (Roche Life Sciences) according to manufacturer's instructions. Prepared cDNA (20 μL) was diluted with RNase‐free water to a total volume of 100 μL, and 5 μL of this solution was employed for qRT‐PCR using SYBR Green Quantitative Kit (Sigma‐Aldrich). The specificity of the qRT‐PCR was checked by melting curve analysis, and the relative levels of transcription were calculated using the $2^{-\Delta \Delta C_{T}}$ method. The list of primers is provided in Table S2.

### 2.12 5‐Aza treatment and wound‐healing assay

Prior to 5‐Aza treatment, its dose–response curves were obtained using the MTT assay performed in accordance with our previous study (Heger *et al*., 2016a). We chose the optimal concentration that allowed the cells to tolerate the treatment without affecting proliferation (Fig. S1). For the wound‐healing assay, the cells were cultured in 6‐well plates until they reached ~ 80% confluency. Then, a pin was used to scratch and remove cells from a discrete area of the confluent monolayer to form a cell‐free zone. The cells were then treated with sarcosine (1 μm), DNA‐hypomethylating agent, 5‐Aza (1 μm) and their combination (1 μm sarcosine and 1 μm 5‐Aza) and incubated up to 24 h. At 6, 12 and 24 h, micrographs of the cells were taken using the EVOS FL Auto Cell Imaging System (Thermo Fisher Scientific) and compared with micrographs obtained at 0 h using tscratch software (CSElab, Zurich, Switzerland).

### 2.13 Clonogenic assay

Cells were seeded in a 6‐well plate at a density of 1 × 10^4^ cells per well in growth medium and incubated for 6 h. Then, the cells were treated with 1 μm 5‐Aza, 1 μm sarcosine and their combination (1 μm sarcosine and 1 μm 5‐Aza, 24 h) as indicated. After medium renewal, the cells were incubated 8 days in adequate treatment‐free culture media. Finally, the cells were washed with PBS and fixed using 500 μL of 3 : 1 MeOH : acetic acid for 5 min. To visualize the colonies, the cells were stained using 500 μL of 0.5% crystal violet in MeOH for 15 min.

### 2.14 Western blotting

Total cellular proteins were extracted with 100 μL of radioimmunoprecipitation assay buffer containing protease inhibitor cocktail. After electrophoresis, the proteins were electrotransferred onto a nitrocellulose membrane that was blocked with 5% (w/v) nonfat dry milk for 1 h at 37 °C. The membranes were incubated with primary mouse anti‐Dnmt1 (dilution 1 : 2000), rabbit anti‐Dnmt3a (1 : 500), rabbit anti‐Dnmt3b (1 : 500) or mouse anti‐GAPDH (1 : 700, all purchased from Thermo Fisher Scientific) overnight at 4 °C. After washing, the membranes were incubated with anti‐mouse or anti‐rabbit horseradish peroxidase (HRP)‐conjugated secondary antibodies (Dako, Glostrup, Denmark) for 1 h at 20 °C. Proteins were detected using Clarity Western ECL substrate (Bio‐Rad, Hercules, CA, USA) and visualized using the Azure c600 imager (Azure Biosystems, Dublin, CA, USA).

### 2.15 Preparation of prostate tissue specimens

Following Institutional Review Board approval, we accessed prostate tissue from the Department of Pathology, University Hospital Brno. The samples included four prostate tissue specimens (1 benign only, 3 malignant mixed with benign tissue) that had been obtained from prostatectomies and transurethral resections of subjects with signed informed consent. Tissues were fixed in 10% buffered formalin and embedded in paraffin. A 1–4 μm section of each tissue sample was mounted on glass slides and stained with haematoxylin–eosin (H&E). A pathologist (E.T.) reviewed and annotated normal and cancerous areas on the H&E‐stained adjacent tissue sections. Then, 10‐μm‐thick adjacent sections were obtained using Leica SM2010 R (Leica, Wetzlar, Germany). These tissue sections were mounted on glass microscope slides for immunohistochemistry (IHC) and desorption electrospray ionization (DESI) mass spectrometry imaging (MSI) or indium tin oxide‐coated slides for matrix‐assisted laser desorption/ionization time‐of‐flight (MALDI‐TOF) MSI. The study conformed to the standards set by the Declaration of Helsinki.

### 2.16 IHC of prostate tissue sections

Tissue sections (10** **μm) were deparaffinized with xylene and rehydrated in a graded ethanol series of decreasing ethanol concentrations. Heat‐induced epitope retrieval was performed using sodium citrate buffer (10** **mm sodium citrate, 0.05% Tween‐20, pH 6.0) for 20** **min. Sections were blocked with 5% BSA (1** **h) and incubated in anti‐Dnmt1 (1 : 200, in PBS‐T with 1% BSA) overnight at 4 °C in a humidified chamber. To control IHC specificity, primary antibodies were replaced by nonbinding immunoglobulins. After incubation in 0.3% H_2_O_2_ in PBS for 15** **min, the samples were incubated in secondary HRP‐conjugated rabbit anti‐mouse antibody (P0260; Dako) in PBS‐T with 1% BSA for 1 h at 25 °C. Finally, sections were developed with Vector VIP Peroxidase Substrate Kit (Vector Laboratories, Burlingame, CA, USA) at 25 °C for 10** **min, mounted with DPX Mountant for histology and examined using the EVOS FL Auto Cell Imaging System (Thermo Fisher Scientific).

### 2.17 DESI MSI

Desorption electrospray ionization mass spectrometry imaging was performed using the mass spectrometer OrbiTrap Elite (Thermo Fischer Scientific) with a DESI‐2D ion source (Prosolia, Indianapolis, IN, USA). Imaging experiments were performed by continuous scanning of the tissue surface with spraying liquid (8 : 2, MeOH/water, v/v) at a flow rate 2 μL·min^−1^, scanning velocity of 65 μm·s^−1^ and a 55° spray impact angle in the positive ion mode (for sarcosine *m/z,* 90.0549). The obtained data were processed using the BIOMAP 3.8.0.3 software (Novartis Institutes for Biomedical Research, Cambridge, MA, USA) to create 2D ion images.

### 2.18 MALDI‐TOF MSI

The MSI was performed on a MALDI‐TOF/TOF mass spectrometer Bruker UltrafleXtreme (Bruker Daltonik GmbH). As MALDI matrix, we used 2,5‐dihydroxybenzoic acid (30** **mg·mL^−1^) in MeOH/water (50 : 50, v/v) with 0.2% trifluoroacetic acid. Prior to analyses, tissue sections were scanned and loaded into fleximaging 3.0 software (Bruker Daltonik GmbH). The *m/z* images were generated and visualized using scils lab 2014b software (Bruker Daltonik GmbH). The detection of SAMe and SAH was confirmed by MALDI‐TOF/TOF analysis using LIFT cell by detecting typical fragments of the SAMe precursor ion at *m/z* 399.145 and SAH precursor ion at *m/z* 385.130. Typical fragment ions of SAMe were identified at *m/z* 250.095, 136.063 and 102.055, whereas typical fragment ions of SAH were at *m/z* 250.044, 136.074 and 134.009.

### 2.19 Descriptive statistics

For the statistical evaluation of the results, the mean was taken as the measurement of the main tendency, while the standard deviation was taken as the dispersion measurement. Differences between groups were analysed using a paired *t*‐test. For analyses, Software statistica 12 (StatSoft, Tulsa, OK, USA) was employed.

## 3 Results

### 3.1 Incubation of prostate cells with sarcosine elicits the upregulation of the sarcosine N‐demethylation enzymes

**Figure 2:**
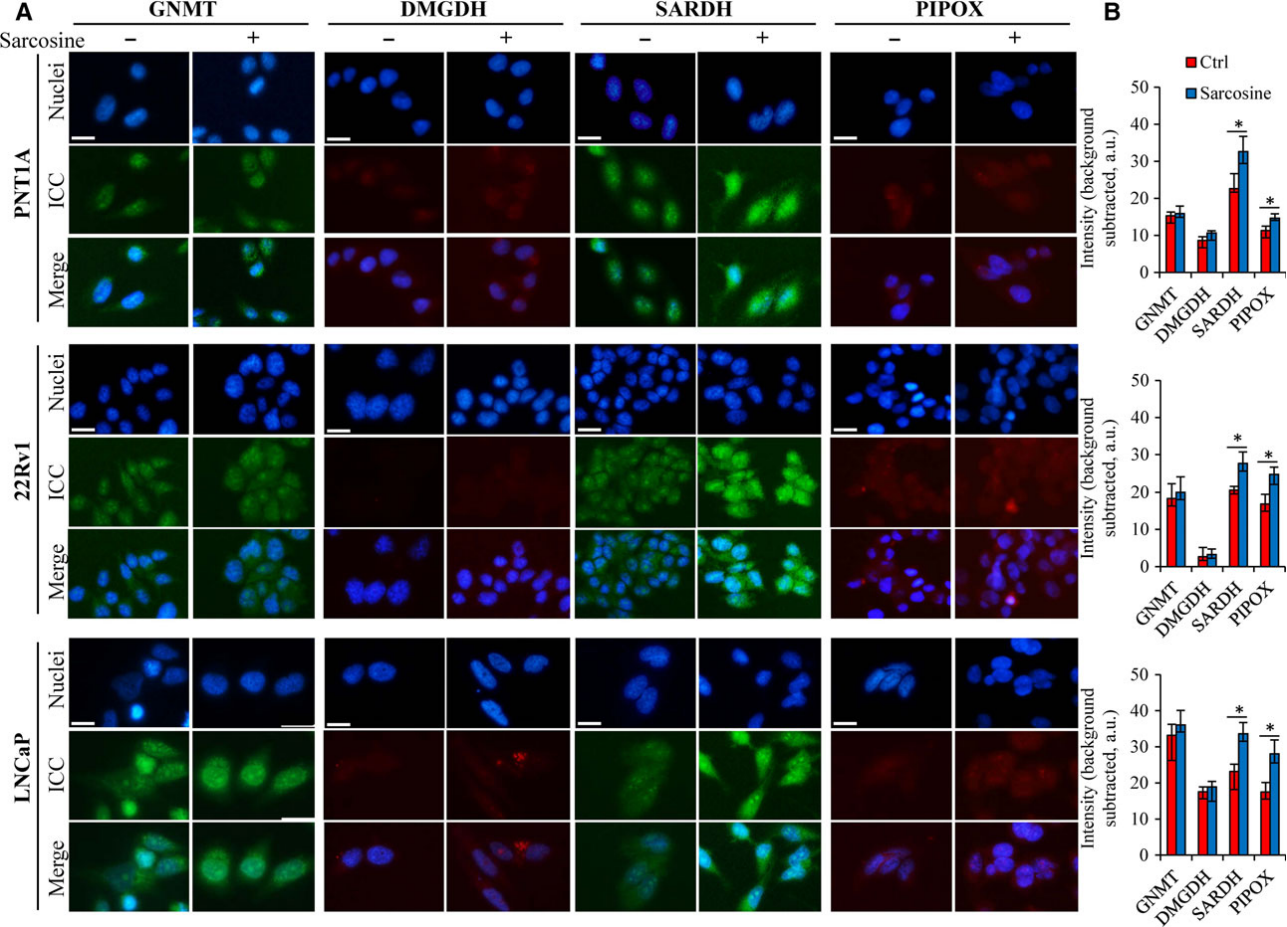
Sarcosine‐related effects on the expression of enzymes involved in its metabolic pathway. (A) Representative ICC of sarcosine metabolic‐related enzymes in prostate cells incubated without (left panel) or with (right panel) sarcosine (1 μm, 24 h). Nuclei were counterstained with Hoechst 33342. Images shown are representative of those obtained from three independent experiments. The length of the scale bar is 50 μm. (B) Quantification of ICC of the analysed enzymes performed using imagej software (National Institutes of Health, Bethesda, MD, USA). The bars show the medians of five (*n *=* *5) independent replicates for 80–120 cells per group. Background, assessed without primary antibody, has been subtracted. The vertical bars indicate the standard deviation. The data with asterisks (*) indicate significance (*P *<* *0.05, paired *t*‐test) compared with nontreated cells.

We first confirmed our earlier finding that sarcosine stimulates the expression of SARDH but not GNMT (Heger *et al*., 2016a). Additionally, we examined the expression of DMGDH, which is involved in the subpathway that synthesizes sarcosine from dimethylglycine, and PIPOX, which catalyses the oxidative demethylation of sarcosine to yield glycine. Figure 2A,B illustrates that sarcosine incubation had significant (*P *<* *0.05) stimulatory effects on the cytoplasmic expression of SARDH and PIPOX. In contrast, no effect on GNMT and DMGDH, which participate in sarcosine synthesis, was identified. Together with HPLC‐FLD that revealed a rapid intracellular accumulation of sarcosine (Table S3), the obtained data indicate that the addition of sarcosine increases its intracellular pool, which leads to the consequent upregulation of SARDH and PIPOX, enzymes mediating sarcosine N‐demethylation.

### 3.2 Sarcosine alters the global methylation status of prostate cells

**Figure 3:**
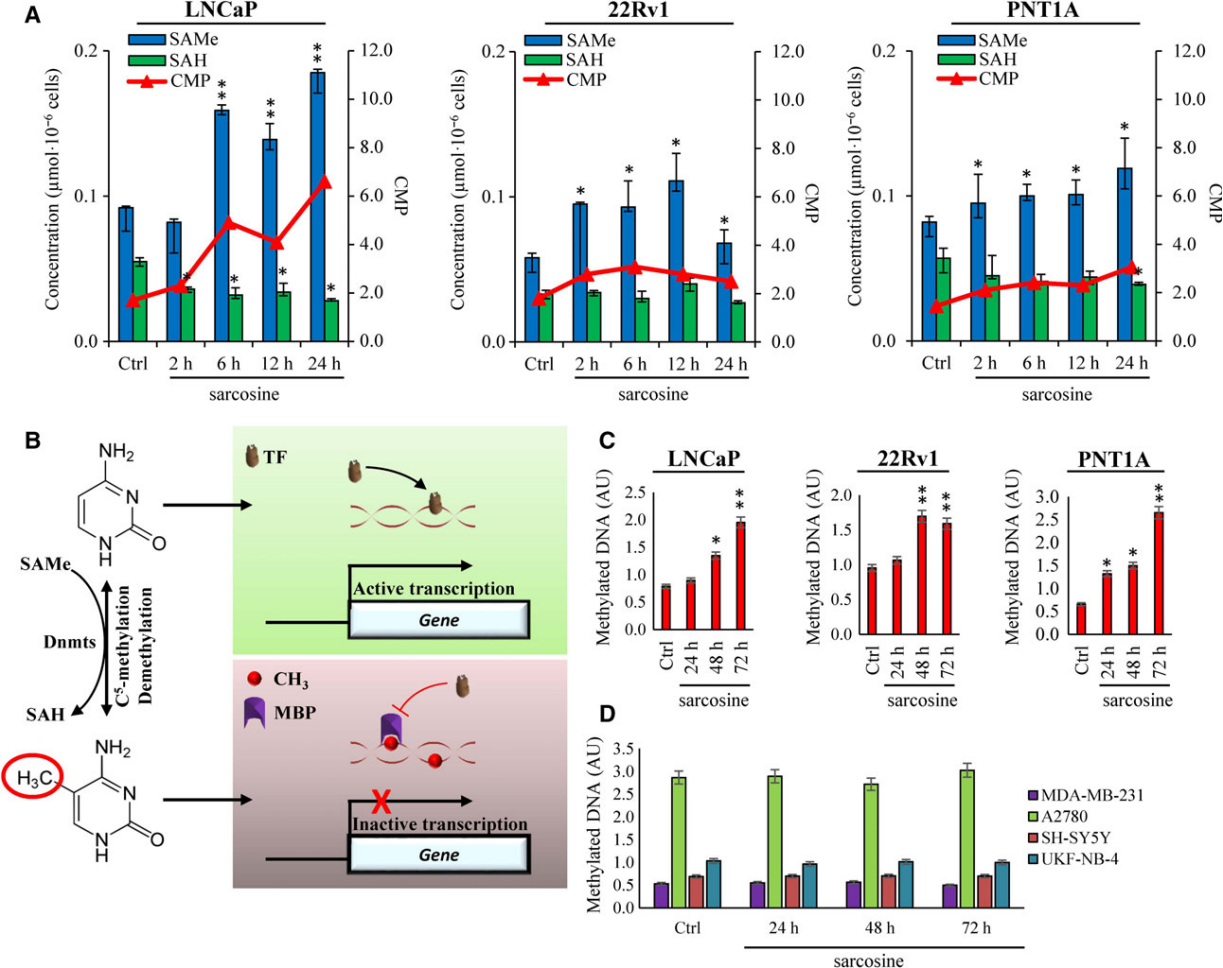
Sarcosine treatment has stimulatory effects on the methyl‐donor SAMe and global methylation status of prostate cells. (A) Values of SAMe and SAH and their ratios (CMP, cellular methylation potential) in PNT1A, 22Rv1 and LNCaP cells nontreated and treated with sarcosine (1 μm). (B) Schematic depiction of roles of SAMe and SAH in C5‐methylation of cytosine to 5‐methylcytosine and the consequent effects on gene transcription. TF, transcription factor; MBP, methyl‐CpG binding proteins. Global methylation indices of (C) prostate and (D) nonprostate cell lines treated with sarcosine (1 μm). Methylation indices were analysed using the MDQ1 Imprint Kit. The values are expressed as the median of three replicates (*n *=* *3) obtained from five independent experiments (*n *=* *5). Ctrl: cells analysed upon 24‐h incubation in medium without addition of sarcosine. The vertical bars indicate the standard deviation. *P < 0.05, **P <* *0.01 (paired *t*‐test) compared with Ctrl – nontreated cells.

As we identified stimulatory effects of sarcosine on the expression of sarcosine‐converting enzymes, we continued with the quantification of SAMe and SAH. Figure 3A illustrates that in all prostate cell lines, sarcosine induced a significant elevation of SAMe. On the other hand, the amount of SAH was either decreased (metastatic LNCaP) or negligibly affected (nonmalignant PNT1A and malignant 22Rv1). This finding was also reflected by a significant increase in cellular methylation potentials (CMP, also SAMe : SAH ratio) (Ulanovskaya *et al*., 2013), particularly in metastatic LNCaP cells. In mammals, > 90% of SAMe is used for methylation reactions; therefore, in the next step, we focused on evaluating the extent of DNA methylation (schematized in Fig. 3B).

To understand the role of sarcosine in the DNA methylation, we first analysed time‐course changes in global methylation of genomic DNA isolated from prostate cells (Fig. 3C). The highest increase in global methylation was identified for PNT1A cells, which also had the lowest relative baseline methylation. A similar trend was found in LNCaP and 22Rv1 cells, for which a faster onset of DNA methylation (upon 48‐h incubation with sarcosine) was identified. We further examined the specificity of sarcosine‐induced methylation in prostate cells. Figure 3D demonstrates that sarcosine failed to enhance DNA methylation in all tested nonprostate cells.

As SAMe is also involved in the biosynthesis of polyamines, we examined the effect of sarcosine on the intracellular pool of Spm and Spd. Figure S2 illustrates no significant contribution of the sarcosine‐to‐SAMe axis towards Spm and Spd levels. Overall, our results indicate a certain connection between sarcosine metabolism and the DNA methylation processes of prostate cells.

### 3.3 Sarcosine elicits the upregulation of Dnmts and promotes aberrant promoter methylation patterns

**Figure 4:**
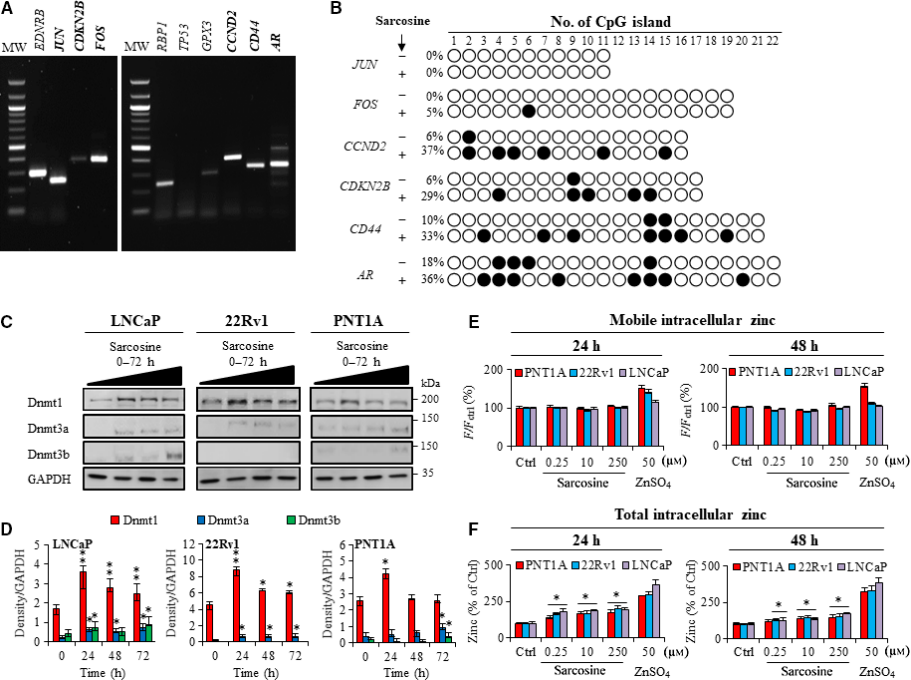
Sarcosine promotes aberrant methylation patterns and stimulates the expression of Dnmts. (A) Representative gels showing the products of BSP. The genes marked in bold were subsequently analysed by Sanger sequencing. MW – weight marker. (B) Methylation patterns of CpG islands of tested promoter regions of genes isolated from LNCaP cells. Empty circles and black circles indicate unmethylated and methylated CpG islands, respectively. The numbers on the right represent levels of methylation. (C) Immunoblots of Dnmt1, Dnmt3a and Dnmt3b in prostate cells treated with sarcosine for 24, 48 and 72 h. (D) Densitometry showing quantification of expression of Dnmts normalized to GAPDH. (E) Quantification of pool of intracellular mobile zinc using Zinpyr‐1 and (F) intracellular total using AAS. The values are expressed as the median of three replicates (*n *=* *3) obtained from five biological replicates (*n *=* *5). The vertical bars indicate the standard deviation. *P <* *0.05, **P <* *0.01 (paired *t*‐test) compared with Ctrl – nontreated cells.

Significant stimulation of global DNA methylation prompted us to investigate the methylation patterns of CpG‐rich islands in promoter regions of selected genes crucial for PCa development and progression. BSP sequencing (BSP product validation is shown in Fig. 4A) revealed that sarcosine caused a denser promoter methylation, particularly in *CCND2* (6% vs. 37% postsarcosine treatment), *CDKN2B* (6% vs. 29%), *CD44* (10% vs. 33%) and androgen receptor (*AR*) (18% vs. 36%). In contrast, no significant contribution of sarcosine towards the methylation of promoters of two analysed proto‐oncogenes (*JUN* and *FOS*) was found (Fig. 4B). Notably, a sarcosine‐induced decrease in transcriptional activity of *CCND2* and *CDKN2B* was identified in our previously published microarray‐based study (Merlos Rodrigo *et al*., 2017).

Methyl groups are catalytically transferred from SAMe to DNA by enzymes from the Dnmt family. Therefore, we investigated the influence of sarcosine on the expression of the three major Dnmts (Fig. 4C). In all prostate cells, sarcosine caused a pronounced stimulation of Dnmt1 (Fig. 4D). Interestingly, after 24 h, Dnmt1 expression decreased. On the other hand, the expression of Dnmt3a and Dnmt3b was stimulated upon longer sarcosine incubation. This result could be connected to divergent roles of Dnmts and their distinct methylation activities.

As Dnmts contain zinc finger structural motifs that coordinate zinc ions to stabilize the protein fold (Frauer *et al*., 2011; Otani *et al*., 2009), we further estimated the influence of sarcosine on levels of mobile vs. total intracellular zinc. Notably, sarcosine did not affect the intracellular mobile zinc pool (Fig. 4E). In contrast, total intracellular zinc status was markedly increased in all prostate cell lines upon incubation with sarcosine (Fig. 4F). This finding also corresponds to Dnmt1 expression. However, due to the abundance of zinc finger motifs within the human proteome, the stimulatory effects of sarcosine on zinc homeostasis should be further studied in detail.

### 3.4 Blunting of Dnmts by 5‐Aza inhibits sarcosine migration and proliferation

**Figure 5:**
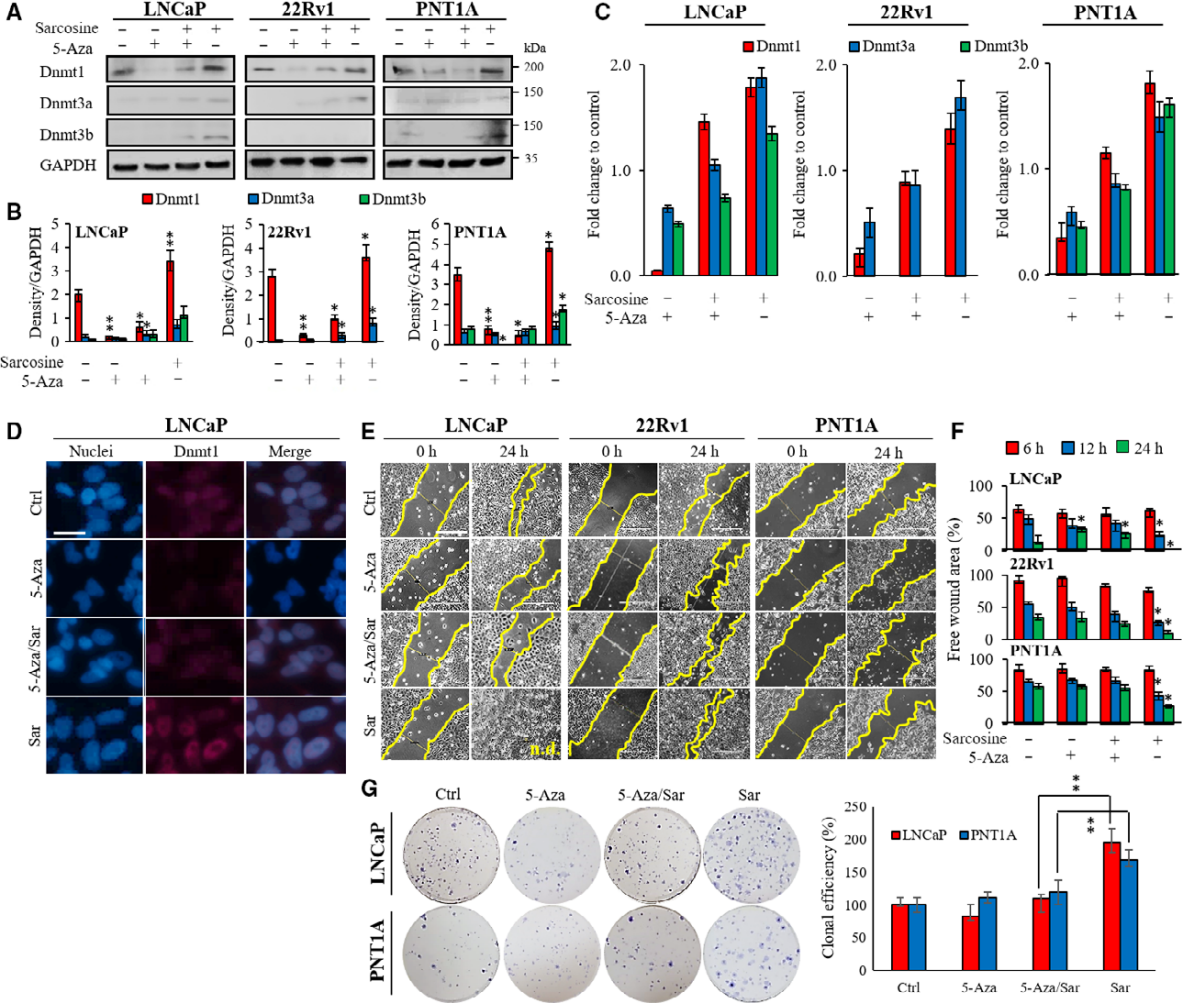
The hypomethylating agent 5‐Aza impairs sarcosine‐induced stimulation of Dnmts and cell migration. (A) Immunoblots of Dnmt1, Dnmt3a and Dnmt3b in prostate cells treated with 5‐Aza, sarcosine and their combination (24 h). (B) Densitometry showing quantification of expression of Dnmts normalized to GAPDH. (C) Fold change expression of Dnmts‐encoding mRNA analysed by qRT‐PCR. (D) ICC of DNMT1 in prostate metastatic cells (LNCaP) upon treatment with 5‐Aza, sarcosine and their combination. Scale bar, 30 μm. (E) Cell migration evaluated by monolayer wound‐healing assay. Scale bar, 500 μm. (F) The wounds were quantified at different time points using tscratch software. All values are expressed as the median of five biological replicates (*n *=* *5). (G) Clonogenic assay conducted with the treatment of sarcosine, 5‐Aza and their combination as described in Materials and methods. *Left*: the full view of wells stained with crystal violet. *Right*: the quantification of clonal efficiency. The results are represented as the median of five independent experiments (*n *=* *5). The vertical bars indicate the standard deviation. *P <* *0.05, **P <* *0.01 (paired *t*‐test).

To verify that DNA methylation plays a role in the sarcosine‐induced stimulation of prostate cells, we first assessed the effects of 5‐Aza, a pharmacological DNA methylation inhibitor on sarcosine‐induced expression of Dnmts. We showed that in all prostate cells, 5‐Aza treatment (10 μm) could diminish Dnmt1 expression (Fig. 5A) without noticeable toxicity (Fig. S1). Identification of the inhibitory effects of 5‐Aza on Dnmt3a and Dnmt3b was biased by their low basal expression (Fig. 5B). Hence, we validated the experiment using qRT‐PCR. Figure 5C confirms the pronounced inhibitory activity of 5‐Aza. Interestingly, sarcosine partially reverted 5‐Aza activity, particularly for Dnmt1. This finding was confirmed by ICC demonstrating nuclear localization of Dnmt1 and its slightly reverted expression upon 5‐Aza/sarcosine treatment (Fig. 5D). To further address whether 5‐Aza might regulate the previously described sarcosine‐induced migration (Heger *et al*., 2016a; Khan *et al*., 2013; Sreekumar *et al*., 2009), we next analysed the effects of the above‐discussed treatments using the monolayer wound‐healing assay. As shown in Fig. 5E, sarcosine caused a considerable increase in migration of prostate cells with the lowest stimulatory effects identified for PNT1A cells. Moreover, the depletion of Dnmt by 5‐Aza followed by sarcosine treatment resulted in pronounced inhibition of sarcosine‐induced migration (quantified in Fig. 5F). Likewise, 5‐Aza demonstrated inhibitory activity towards sarcosine‐induced clonogenic growth (Fig. 5G). Overall, our data confirmed that phenotypic properties of prostate cells can be markedly influenced by cross talk between epigenetics and sarcosine metabolism.

### 3.5 Spatial mapping reveals concurrence in content of sarcosine, SAMe and Dnmt1 in histologically confirmed malignant zones

**Figure 6:**
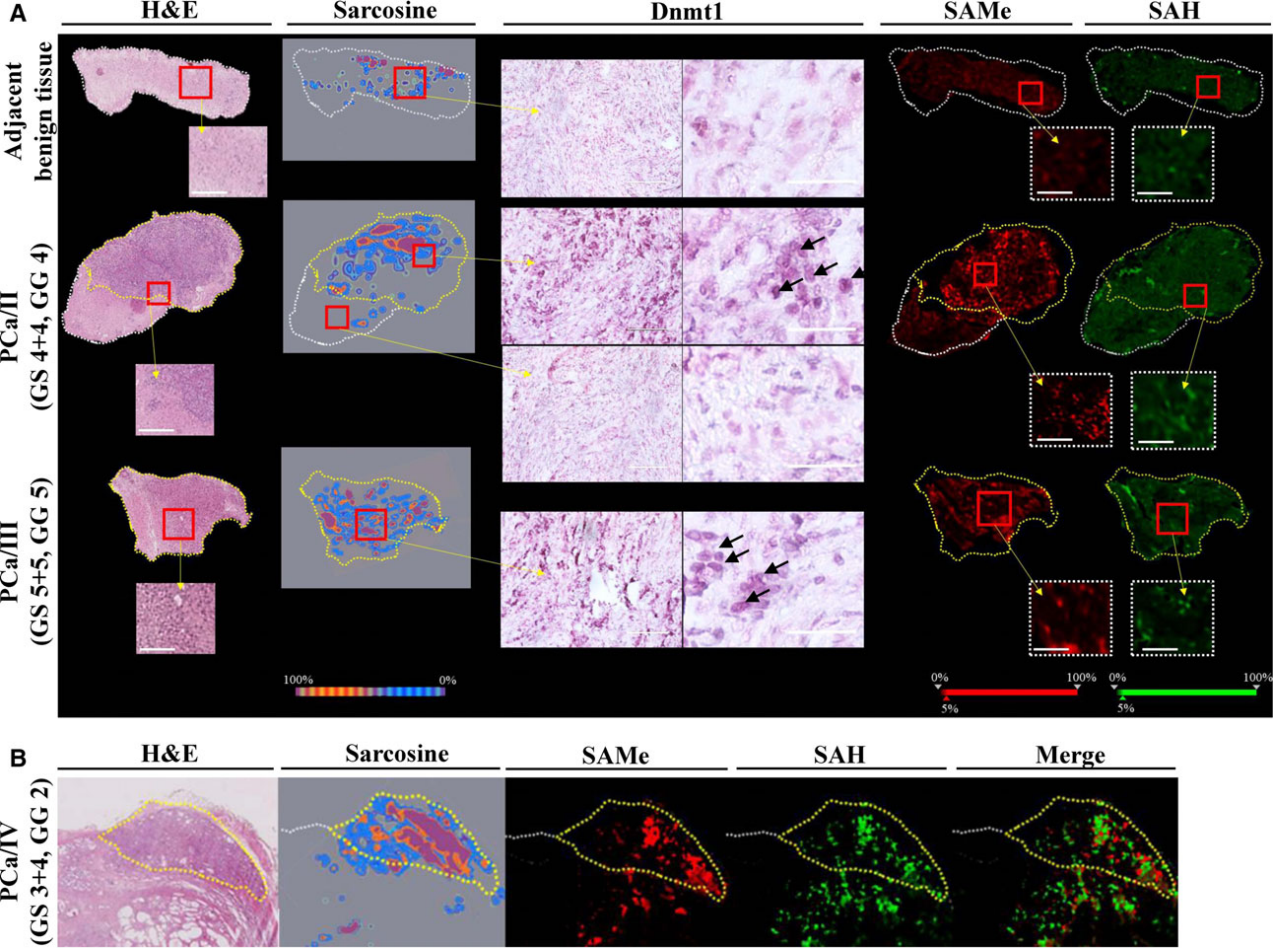
MALDI‐TOF MSI and DESI MSI of formalin‐fixed paraffin‐embedded prostatic tissue sections. (A) The representative MALDI‐TOF and DESI MSI 2D molecular images are shown together with the corresponding H&E staining with yellow demarcated malignant zones and IHC of Dnmt1 showing its high nuclear expression in malignant specimens. Adjacent benign tissue – prostate tissue without confirmed malignancy adjacent to histologically confirmed PCa zone, PCa/II – prostate acinar adenocarcinoma [Gleason score (GS) 4 + 4, Gleason grade (GG) 4], PCa/III – prostate acinar adenocarcinoma (GS 5 + 5, GG 5). Enlarged are representative views. Scale bar, 100 μm. Arrowheads indicate nuclei with high expression of Dnmt1. (B) Distribution of SAMe and SAH vs. sarcosine in a representative (PCa/IV, GS 3 + 4, GG 2) tissue specimen showing significant elevation of SAMe and sarcosine but only slight elevation of SAH in the histologically confirmed malignant zone. Scale bar, 1 mm. The MSI image of [SAMe+H]^+^ molecular ion (*m/z* 399.145 ± 0.100) has red‐coded ion intensity, while the intensity of [SAH+H]^+^ molecular ion (*m/z* 385.130 ± 0.100) is green coded. H&E‐stained images show defined areas of PCa (yellow dashed line).

To investigate the importance of sarcosine and the SAMe‐Dnmt1 axis in prostate tissues, we performed a spatial mapping of paraffin‐embedded specimens using MSI and IHC. For each case, a 10 μm section taken immediately adjacent to the H&E section was used. As shown in Fig. 6A, analyses were performed on tissue samples with histologically demarcated areas of normal and malignant tissue (yellow dashed line).

We found that malignant zones had considerably higher nuclear expression of Dnmt1 compared with benign tissue. This finding agrees with studies that identified increasing Dnmt1 levels during PCa progression and development of a castration‐resistant phenotype (Chen *et al*., 2010; Patra *et al*., 2002; Valdez *et al*., 2013). Catalytic transfer of methyl groups by Dnmts is closely dependent on the methyl‐donor SAMe. Therefore, using pixel‐to‐pixel MSI spectral data (representative spectra and total ion chromatogram are shown in Fig. S3 and Fig. S4A,B), 2D molecular images were constructed to visualize the spatial distribution of sarcosine, SAMe and SAH in prostate tissues. We identified substantial differences in sarcosine and SAMe levels between benign and malignant tissues. As an upregulation of Dnmt1 and an increased production of SAMe were partially observed in adjacent benign tissues (Fig. 6B), spatial analyses outline a new hypothesis that SAMe could possibly induce changes in the prostate tumour microenvironment. We are eager to further investigate this aspect.

## 4 Discussion

In the present study, we demonstrated that the incubation of prostate cells with sarcosine, a PCa oncometabolite, leads to concurrent increase in formation of SAMe, which caused increased prostate cell‐specific methylation of CpG islands as a result of upregulation of Dnmts, particularly Dnmt1. Our results represent the first evidence that the sarcosine pathway is involved in regulating the prostate epigenetic landscape, highlighting that sarcosine is an important factor underlying PCa pathogenesis.

In cancer, metabolic rewiring modifies the epigenetic landscape via modulating the activities of a wide spectrum of biomolecules, including DNA‐modifying enzymes, miRNA and lncRNA. A number of metabolic alterations have been previously linked to epigenetic aberrations. These aberrations include inactivating mutations of fumarate hydratase, succinate dehydrogenase, isocitrate dehydrogenase and others (Janeway *et al*., 2011; Sciacovelli *et al*., 2016). These events have a common feature, the incremental accumulation of respective oncometabolites, which drives tumorigenesis via epigenetic reprogramming.

Metabolism of sarcosine is vital for PCa. It has been previously reported that the sarcosine‐forming enzyme GNMT is upregulated in localized and metastatic PCa relative to benign tissue and that high GNMT cytoplasmic expression is associated with lower disease‐free survival rates of patients with PCa (Khan *et al*., 2013; Song *et al*., 2011). In contrast, the sarcosine N‐demethylating enzymes SARDH and PIPOX were downregulated in PCa tissues (Khan *et al*., 2013). These events lead to a mechanistic accumulation of sarcosine (Sreekumar *et al*., 2009), which has been found to potentiate PCa progression by stimulating proliferation, invasion and intravasation.

Sarcosine is a known ligand of the proton‐coupled amino acid transporters, by which it can be efficiently taken up (Piert *et al*., 2017). This finding agrees with our data showing fast intracellular accumulation of sarcosine being the highest for metastatic (LNCaP) cells and the lowest for nonmalignant (PNT1A) cells. Although we have not examined the route of uptake, a significant increase in intracellular sarcosine provides clear evidence that prostate cells are capable of taking up sarcosine from the external environment.

The sarcosine N‐demethylation pathway is in close proximity to the methionine cycle, in which SAMe is converted from methionine (Wilson *et al*., 2009). Notably, the intracellular accumulation of sarcosine was accompanied by the stimulation of SAMe production. As discussed *vide supra*, metastatic cells had the highest rate of sarcosine uptake. Interestingly, this phenomenon was associated with a significantly higher increase in SAMe and CMP compared with the rest of the tested cells. Accordingly, it has been previously demonstrated that sarcosine stimulatory activity towards a more invasive phenotype is not only the highest for metastatic cells but also acts in cells derived from benign and malignant prostate tissues through upregulation of genes involved in cell cycle and mitosis, while downregulating genes driving apoptosis (Heger *et al*., 2016a; Khan *et al*., 2013; Merlos Rodrigo *et al*., 2017).

A direct stimulatory effect of sarcosine on SAMe can explain the increase in the aggressiveness of prostate cells. An excess supply of SAMe might contribute to DNA hypermethylation and inappropriate gene silencing. Likewise, SAMe is required for the *de novo* biosynthesis of polyamines, which are essential for cancer cell growth (Nowotarski *et al*., 2013). Surprisingly, in all tested prostate cells, we identified a marked stimulatory effect of sarcosine towards DNA methylation without any significant effect on intracellular Spd and Spm.

It is worth to note that sarcosine did not cause an increase in the DNA methylation of tested cells of nonprostate origin, and thus, the described phenomenon is most likely predominant for prostate cells. Noteworthy, it has been found that human epidermal growth factor‐2 (HER‐2)‐positive breast tumours display upregulation of sarcosine metabolism‐related enzymes (GNMT, SARDH, PIPOX) (Yoon *et al*., 2014). Thus, investigation of linkage between sarcosine and DNA methylation status in these cells might be performed to elucidate importance of sarcosine for HER‐2 positive breast tumours.

We anticipate that sarcosine‐induced prostate‐specific regulation of DNA methylation could be attributed to high intracellular zinc levels, unique to prostate cells. In 1985, Wallwork and Duerre discovered that zinc deficiency *in vivo* is reflected in the depressed rates of global DNA methylation (Wallwork and Duerre, 1985). Interestingly, they also identified that zinc deficiency does not impair the synthesis of SAMe and SAH, but the methyl group of SAMe turned over substantially more slowly in zinc‐deficient rats. Despite this finding, it should also be noted that PCa cells can revert the zinc accumulation phenotype to reach higher levels of citric acid cycle activity (Costello *et al*., 2004). This result can presumably explain substantially lower increments of DNA methylation in malignant and metastatic cells compared with that in their nonmalignant counterparts, as demonstrated in Fig. 3C.

In subsequent experiments, we analysed the promoter methylation of several genes pivotal for prostate tumorigenesis. Importantly, sarcosine induced a higher density of promoter methylation of *CCND2* and *CDKN2B*, which both can inhibit the cell cycle G_1_/S transition. These findings are in line with our previous study showing depressed transcriptional activity of *CCND2* and *CDKN2B* in prostate cells due to sarcosine exposure (Merlos Rodrigo *et al*., 2017). In addition, we also identified significantly denser methylation of the *AR* promoter. Although we did not analyse whether this can result in *AR* transcriptional block, this phenomenon could be responsible for the loss of AR expression and a consequent development of tumour hormonal independence (Kinoshita *et al*., 2000). Based on our pilot data, it is indisputable that genomewide methylation analyses of prostate tissues varying in sarcosine levels by cutting‐edge techniques, such as pyrosequencing, could pronouncedly contribute to understanding the role of sarcosine in PCa pathogenesis.

The aberrant expression of Dnmts and disruption of DNA methylation patterns associated with various types of cancers is well known. For example, Valdez *et al*. (2013) revealed that in PCa, Dnmt1 nuclear staining significantly increased from normal to metastatic cancer. As the only way of methyl group transfer from SAMe to DNA is through catalytic activity of Dnmts, it is not surprising that sarcosine upregulates expression of these enzymes. Unexpectedly, the highest upregulation was found for Dnmt1, whose main role is to maintain the original pattern of DNA methylation in a cell lineage. However, Dnmt1 also shows capabilities for *de novo* methylation of DNA through functional cooperation with Dnmt3a (Fatemi *et al*., 2002). The upregulation of Dnmt1 has been found to cause a more aggressive phenotype of PCa and a transition to hormone resistance (Chen *et al*., 2010; Valdez *et al*., 2013). Unfortunately, none of these studies attempted to examine the levels of sarcosine in PCa tissues. Therefore, it is difficult to compare our data with published reports. Nevertheless, due to pronounced stimulatory effects of sarcosine on the expression of Dnmts and on the migration and clonal efficiency of prostate cells, we suggest the intracellular accumulation of sarcosine as a presumable factor influencing PCa aggressiveness. This suggestion is in line with studies delineating sarcosine as a metabolite that is highly increased during PCa progression to metastasis and elevated in invasive PCa cell lines (Khan *et al*., 2013; Sreekumar *et al*., 2009).

Interestingly, sarcosine can partially revert the inhibitory activity of 5‐Aza, as evidenced by both the mRNA and protein expression of Dnmts. While we cannot offer a suitable explanation for this phenomenon, our data clearly demonstrated that the partial reversal of Dnmt expression does not enable sarcosine to stimulate prostate cell migration and clonogenicity, as found for sarcosine treatments without 5‐Aza. Since 5‐Aza is a known DNA‐damaging agent inducing formation of double‐strand breaks and G_1_/S arrest (Kiziltepe *et al*., 2007), our results suggest that sarcosine is protecting the exposed cells by inducing Dnmt1, a methyl transferase that is crucial for the genome protection and epigenetic control. As sarcosine can trigger the development of chemoresistance to various anticancer agents (Merlos Rodrigo *et al*., 2017), it can be expected that a combination therapy utilizing Dnmt inhibitors and cytostatic drugs could be beneficial for treatment of aggressive PCa. Indeed, a number of studies can be found describing the improvement of anticancer effects of distinct agents by Dnmt inhibitors in castration‐resistant PCa (Festuccia *et al*., 2009; Sonpavde *et al*., 2008).

Using two *in situ* MSI approaches, we attempted to verify our findings in prostate tissue specimens. Our data provided clear evidence that malignant tissue contains unprecedentedly higher levels of sarcosine, SAMe and Dnmt1. Furthermore, we identified quite strict colocalization. Despite a limited number of samples, the results highlight the presumable importance of these molecules for PCa pathogenesis. Future work should focus on analysing a larger and more heterogeneous cohort of prostate tissue specimens. Beyond a deeper understanding of PCa pathogenesis, the validation of our pioneer findings could lead to the development of a novel class of PCa therapeutic agents that selectively target sarcosine N‐demethylating enzymes to decrease their impact on the SAMe‐Dnmt epigenetic axis.

## 5 Conclusions

In conclusion, as several metabolic alterations have already been connected with the altered epigenetic landscape. The results of the present study suggest a novel link between the sarcosine metabolic pathway and SAMe‐Dnmt‐mediated epigenetic modifications. Our study provides a solid base for further investigation that will be focused on finding the specific gene promoters affected by the sarcosine pathway and a consequent effect on their transcriptional activity. Moreover, our study suggests that the sarcosine‐SAMe‐Dnmt1 axis could be involved in the transition of PCa to hormone resistance. We are eager to continue to study this aspect. Despite analysing a limited number of clinical specimens, our pilot data underpin a role for the studied axis in PCa and the importance of large cohort‐based studies. Unfortunately, due to the retrospective nature of our study, we were not able to collect urinary specimens. However, we anticipate that a comparative analysis of sarcosine in fresh urine specimens and tissues together with examining the SAMe‐Dnmt1 axis could be helpful in resolving the uncertain diagnostic potential of urinary sarcosine.

## Conflict of interest

The authors declare no conflict of interest.

## Author contributions

VS, PM, ZL, RG, SK, LV, ET, BK and CK performed the experimental work. OZ, DP and HH contributed to the analysis and representation of data. VA and ZH wrote the manuscript. MS, TE and ZH participated in the design of the study. All authors approved the final version of manuscript.

## Supporting information


**Fig. S1.** Dose‐response curve of 5‐Aza for all tested prostate cell lines. The values are expressed as the mean of five independent replicates (*n *=* *5).
**Fig. S2.** Intracellular amount of spermine (Spm) and spermidine (Spd) in prostate cells incubated with sarcosine.
**Fig. S3.** Representative average positive ion mode MALDI‐TOF mass spectrum derived from pixel‐to‐pixel construction of 2D molecular images showing both SAMe and SAH.
**Fig. S4.** (A) Representative DESI total ion chromatogram of PCa tissue (PCA_II). (B) Fourier transform MS positive spectrum of sarcosine (*m/z* 90.05491 [M+H]^+^, −0.51399 ppm).
**Table S1.** Sequences of the primers used for BSP.
**Table S2.** Sequences of the primers used for qRT‐PCR and primer validation in LNCaP cells.
**Table S3.** HPLC‐FLD quantitation of intracellular sarcosine in prostate cells.
