# Supplementary material for: Sarcosine is a prostate epigenetic modifier that elicits aberrant methylation patterns through the SAMe‐Dnmts axis
Source: Mol Oncol. 2019 Mar 9;13(5):1002–17. doi: 10.1002/1878-0261.12439 (PMC6487735; doi:10.1002/1878-0261.12439)
Supplement: Supplementary file 1 — Fig. S1. Dose‐response curve of 5‐Aza for all tested prostate cell lines. The values are expressed as the mean of five independent replicates (n = 5). Fig. S2. Intracellular amount of spermine (Spm) and spermidine (Spd) in prostate cells incubated with sarcosine. Fig. S3. Representative average positive ion mode MALDI‐TOF mass spectrum derived from pixel‐to‐pixel construction of 2D molecular images showing both SAMe and SAH. Fig. S4. (A) Representative DESI total ion chromatogram of PCa tissue (PCA_II). (B) Fourier transform MS positive spectrum of sarcosine (m/z 90.05491 [M+H]+, −0.51399 ppm). Table S1. Sequences of the primers used for BSP. Table S2. Sequences of the primers used for qRT‐PCR and primer validation in LNCaP cells. Table S3. HPLC‐FLD quantitation of intracellular sarcosine in prostate cells. [file MOL2-13-1002-s001.docx]

**Sarcosine is a prostate epigenetic modifier that elicits aberrant methylation patterns through SAMe-DNMTs axis**

Vladislav Strmiska, Petr Michalek, Zuzana Lackova, Roman Guran, Sona Krizkova, Lucie Vanickova, Ondrej Zitka, Marie Stiborova, Tomas Eckschlager, Borivoj Klejdus, Dalibor Pacik, Eliska Tvrdikova, Claudia Keil, Hajo Haase, Vojtech Adam, Zbynek Heger

| **Gene** | **Abbreviation** | **GenBank Accession no.** | **Primer pair (5´-3´)*** |
| --- | --- | --- | --- |
| Jun Proto-Oncogene | *JUN* | U60581.1 | TGYGGTAGGGGGGAGGGTAGG  TTATCCAACCCRAACTCAACACTTATCTACTACC |
| Fos Proto-Oncogene | *FOS* | K00650.1 | GTTATTTTYGAAATTTTTTATTTTGGGG  CCRACTCAATCTTAACTTCTCAATTACTC |
| Androgen receptor | *AR* | M58158.1 | GAGATTTYGGGGAGTTAGTTTGTTGGGAGAG  AAAACAACCRTCAATCCTACCAAACAC |
| Cyclin D2 | *CCND2* | AF518005.1 | GAGGATYGGGAGGGGAGGAAAG  AAAAAACRTTCCCCTAACCTCC |
| Cyclin-Dependent Kinase Inhibitor 2B | *CDKN2B* | DQ406745.1 | CGTTCGTATTTTGCGGTT  CGTACAATAACCGAACGACCGA |
| CD44 Antigen | *CD44* | AH003670.2 | ATTTTTGYGGGTTGTTTAGTTATAGTTTTTTTTG  AACACCCCRCACCCATCTTACTACC |

**Table S1:** Sequences of the primers used for BSP.

* Upper and lower sequences represent forward and reverse primers, respectively.

**Table S2:** Sequences of the primers used for qRT-PCR and primer validation in LNCaP cells.

| **Gene** | **Abbreviation** | **GenBank Accession no.** | **Primer pair (5´-3´)*** | **Amplicon size (bp)** |
| --- | --- | --- | --- | --- |
| DNA Methyltransferase 1 | Dnmt1 | NM_001130823.2 | GAGCCACAGATGCTGACAAA  TGCCATTAACACCACCTTCA | 238 |
| DNA Methyltransferase 3a | Dnmt3A | NM_175629.2 | AGCCCAAGGTCAAGGAGAT  CAGCAGATGGTGCAGTAGGA | 232 |
| DNA Methyltransferase 3b | Dnmt3B | NM_175629.2 | CAGGAGACCTACCCTCCACA  TGTCTGAATTCCCGTTCTCC | 242 |
| Glyceraldehyde-3-phosphate dehydrogenase | GAPDH | NM_002046.5 | CAGGAGACCTACCCTCCACA  TGTCTGAATTCCCGTTCTCC | 206 |

* Upper and lower sequences represent forward and reverse primers, respectively.


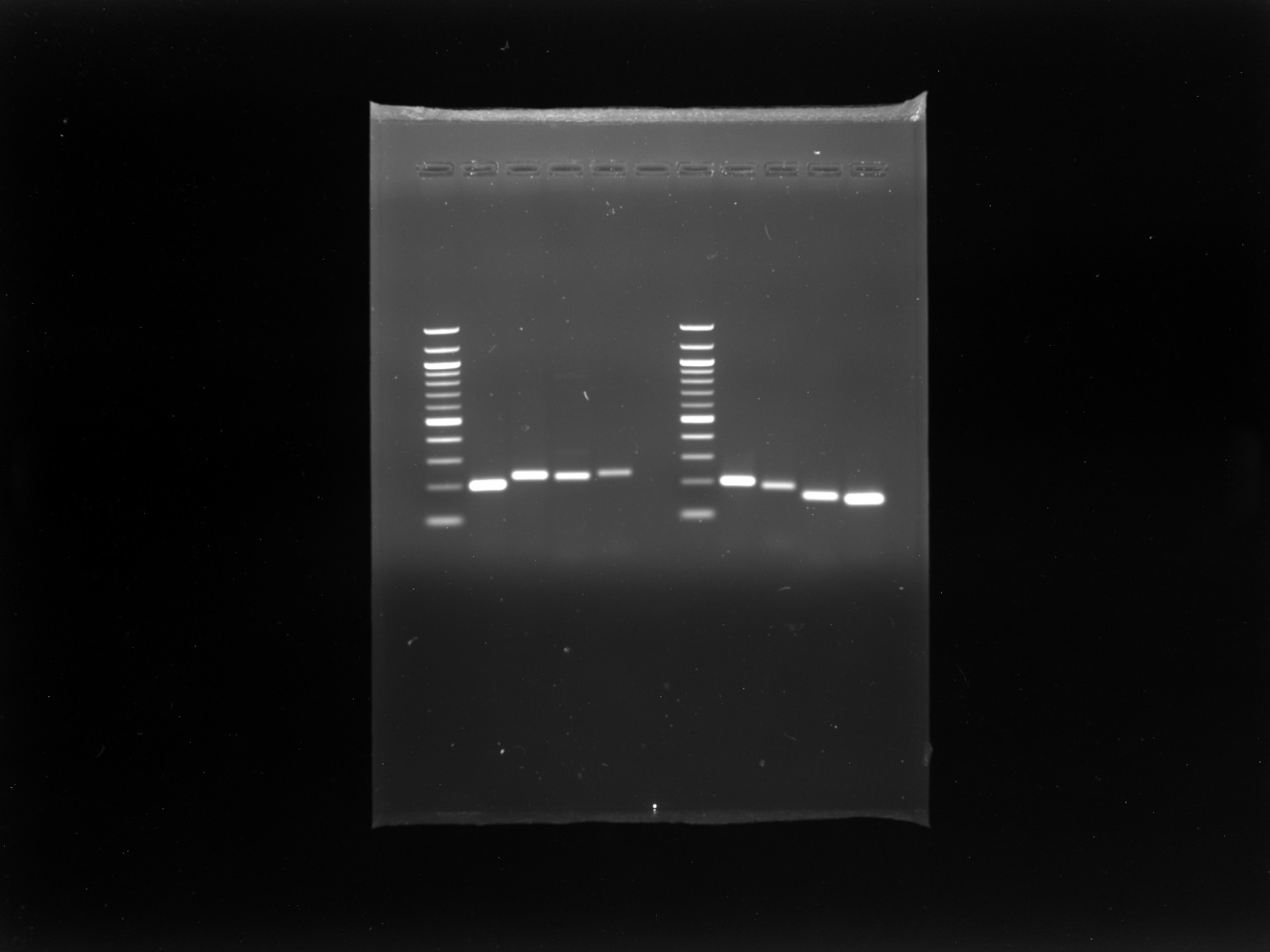


Weight marker / bp

GAPDH / 206

Dnmt1 / 238

Dnmt3a / 232

Dnmt3b / 242

Lane 1

Lane 2

Lane 3

Lane 4

Lane 5

1

2

3

4

5

**Primer validation**

**LNCaP**

95°C/30 s

59°C/30 s

72°C/20 s

30 cycles


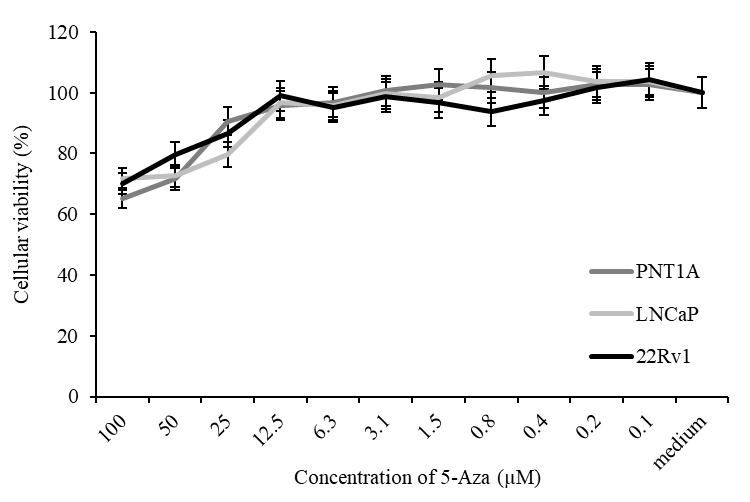


**Fig. S1:** Dose-response curves of 5-Aza for all tested prostate cell lines. The values are expressed as the mean of five independent replicates (*n* = 5). The vertical bars indicate the standard deviation.

**Table S3:** HPLC-FLD quantitation of intracellular sarcosine in prostate cells.

| **Cell line** | **Time point**  **(h)** | **Intracellular sarcosine**  **(nmol/10^6^ cells)** | ***S.D.** |
| --- | --- | --- | --- |
| **PNT1A** | 0 | 0.065 | 0.003 |
|  | 1 | 0.123 | 0.005 |
|  | 3 | 0.110 | 0.004 |
| **22Rv1** | 0 | 0.078 | 0.002 |
|  | 1 | 0.195 | 0.001 |
|  | 3 | 0.221 | 0.005 |
| **LNCaP** | 0 | 0.112 | 0.004 |
|  | 1 | 0.225 | 0.006 |
|  | 3 | 0.199 | 0.002 |

*S.D. - standard deviation


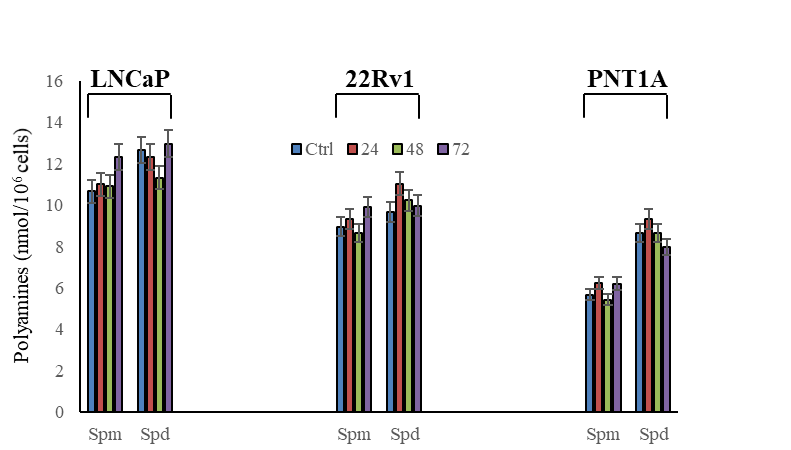


**Fig. S2:** Intracellular amount of spermine (Spm) and spermidine (Spd) in prostate cells incubated with sarcosine. The vertical bars indicate the standard deviation.


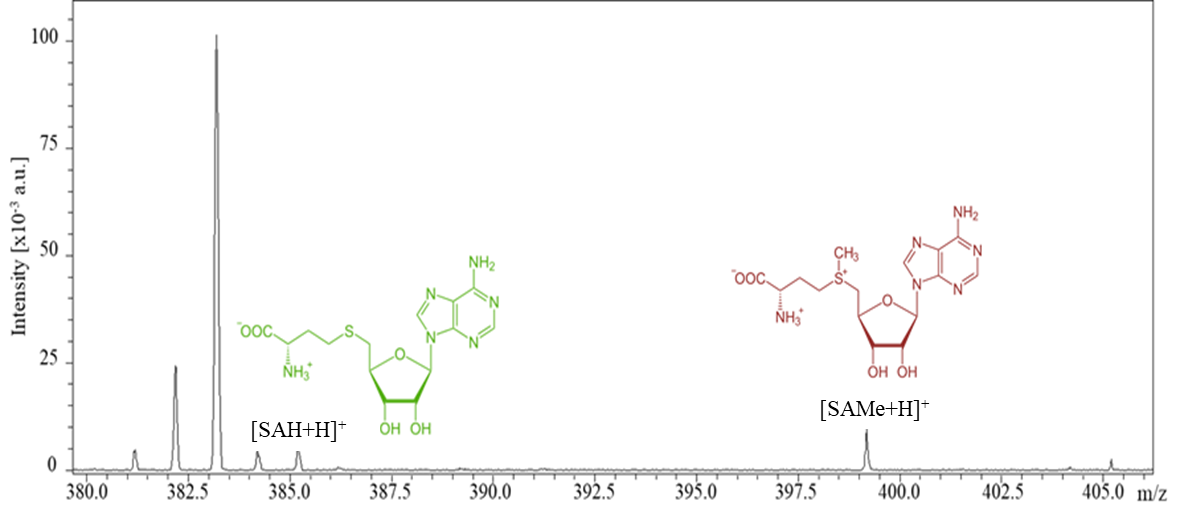


**Fig. S3:** Representative average positive ion mode MALDI-TOF mass spectrum derived from pixel-to-pixel construction of 2D molecular images showing both SAMe and SAH.


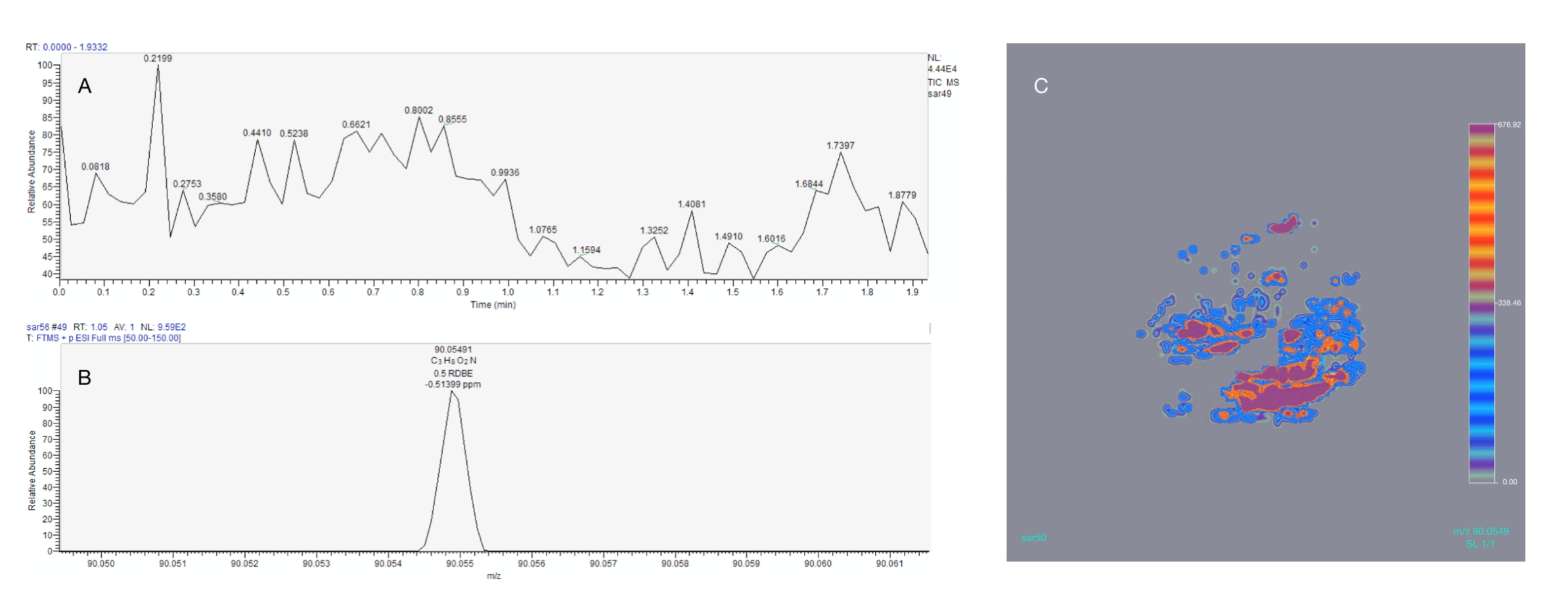


**Fig. S4:** (**A**) Representative DESI total ion chromatogram of PCa tissue (PCa_II). (**B**) Fourier transform MS positive spectrum of sarcosine (*m/z* 90.05491 [M+H]^+^, -0.51399 ppm).
